# Using the Carers’ Alert Thermometer tool to identify needs and support family caregivers of people with motor neurone disease: moving beyond needs assessments

**DOI:** 10.1177/26323524241228306

**Published:** 2024-02-11

**Authors:** Samar M. Aoun, Mary R. O’Brien, Katherine Knighting

**Affiliations:** University of Western Australia and Perron Institute, 8 Verdun Street, Perth, WA 6009, Australia; Faculty of Health, Social Care and Medicine, Edge Hill University, Ormskirk, Lancashire, UK; Faculty of Health, Social Care and Medicine, Edge Hill University, Ormskirk, Lancashire, UK

**Keywords:** amyotrophic lateral sclerosis, caregivers, Carers’ Alert Thermometer, compassionate communities, MND Associations, motor neurone disease, needs assessment, palliative care, support needs

## Abstract

**Background:**

Family caregivers of people with motor neurone disease (MND) experience adverse health outcomes as a result of their caregiving experience. This may be alleviated if their support needs are identified and addressed in a systematic and timely manner. The objective of this pilot study was to assess the feasibility and relevance of the Carers’ Alert Thermometer (CAT) in home-based care, from the perspective of MND family caregivers. The tool provides a formal structure to facilitate discussions with caregivers to enable needs to be addressed.

**Methods:**

This mixed-method study was conducted in Western Australia (2020–2021). Forty-one caregivers and five MND Advisors participated in trialling the CAT intervention which consisted of two encounters with Advisors (6–8 weeks apart) to identify and address support needs through action plans. Caregivers’ feedback was obtained *via* telephone interviews and a thematic analysis was undertaken.

**Results:**

Thirty caregivers completed two CAT assessments. Caregivers identified support priorities of managing their feelings and worries, providing emotional or spiritual care, information about the person’s condition and how their care needs might change. Seventeen caregivers were interviewed and found that this assessment process adequately addressed their needs and it should be continued, it brought the focus onto them to clarify problems and work through solutions. The improvements that were suggested by them, including better information/education in palliative care, led to the development of an online support/information toolkit, which served to empower caregivers and staff by accessing relevant information and resources.

**Conclusions:**

The CAT demonstrated utility for triaging caregivers most in need of additional support and those whom signposting to additional information and self-directed access to support was most appropriate. For any tool to become an integrated part of care, service provider support is key for implementation, allowing for the time resource required and an appropriate education and support structure. MND Associations have an important role in building stronger partnerships with supportive community networks, through compassionate communities models of care, to address the identified needs of MND families in a more sustainable and wholistic manner. Needs assessment is a means towards building this capacity between formal and informal networks.

## Introduction

Motor neurone disease (MND) is a terminal neurodegenerative condition, which results in voluntary muscle weakness, causing loss of limb function and problems with speech, swallowing and respiration.^1^ Death typically occurs within 3 years of symptom onset, usually as a result of respiratory failure.^2^ The effects of the disease are such that there is a high level of dependency on family caregivers who often describe their caring experiences as unrelenting due to the progressive nature of the disease and the hopelessness of recovery.^3–5^ Most individuals with MND live at home, where their psychosocial functioning is intimately connected to the extent and quality of support they receive from family caregivers. Numerous family caregivers spend as many as 15 h a day in their caring role^6^ with the consequent physical and emotional cost known to have a negative impact on their quality of life. Yet, despite this, family caregivers rarely seek help for themselves and infrequently consider their own needs.^7,8^

The National Institute for Health and Care Excellence^9^ in the United Kingdom issued MND-specific guidelines emphasizing the importance of psychological and social support provision for family caregivers. The World Health Organization^10^ also includes caring for family caregivers within its definition of palliative care. One of the cornerstones of a palliative approach to MND care is support for family caregivers.^11,12^ The importance of this area has been validated by a community survey where 84% of respondents (people with MND and their family caregivers *n* = 567) ranked palliative care and support for family members as a strong research priority.^13^ The challenges to achieving high-quality end-of-life care for this group include high levels of caregiver burden; financial and psychological distress; limited respite options; the insufficient integration of services across health and social care; poor and unequal access to coordinated palliative care; significant gaps in the knowledge base of the workforce and a failure to meet the consumer expectations of person-centred care.^12,14^ Recent investigations of MND caregiver support needs after bereavement revealed concerning gaps between what is required and what is received. A large national survey of bereaved MND caregivers found that approximately 40% did not feel their support needs were being met by their health professionals and support services available to them.^15^ A majority of participants (63%) required bereavement support beyond their family and social networks.^16^

MND caregivers’ needs before and after bereavement are largely overlooked as no organization is systematically assessing and addressing their support needs as part of standard practice. Barriers to inclusion in routine practice include: inconsistent identification of caregivers within the care setting; demographic and contextual data on who the caregiver is and their situation; lack of a protocol for assessing caregivers and responding to the assessment; lack of a recording system for caregiver information, separate from patient data.^17^ The implications of not identifying and addressing caregivers’ needs may have serious implications for individuals and wider society as studies have shown that caregivers of people with MND may be at greater risk of developing Prolonged Grief Disorder, post-bereavement, than the general population.^16,18^

Interventions to reduce caregiver burden and distress related to MND have been reported with varying success.^19,20^ Therefore, it is important to design and evaluate effective interventions and find ways to deliver them to families living and caring for someone with MND.^3,21,22^ Service providers in previous studies, that trialled assessment tools without a scoring system, have recommended using an assessment tool with a scoring system to clearly identify the severity of need. This can facilitate timely reviews by alerting staff to those most in need and ensure that resources are appropriately prioritized and directed.^23^

The Carers’ Alert Thermometer (CAT) is an evidence-based triage tool with a scoring system designed to identify and support the needs of family caregivers of people with palliative or progressive conditions.^24^ The CAT has 11 questions. It begins with a general question about any current needs, followed by nine support need indicator questions across two domains of (i) the current caring situation and (ii) the health and well-being of the caregiver. For each indicator question, the caregiver provides a rating for their level of need based on a traffic light system of green (low), amber (medium) and red (high) to identify the level of risk each alert poses to the caregiving situation, and an overall score is calculated. There is an optional question about the end-of-life care preferences of the person being cared for to be used if appropriate. A final resilience question asks caregivers to rate their perceived ability to continue providing care on a five-point scale. The tool has a guidance section of next steps for any needs identified, along with space for an action plan. The final section has space to note an agreed review date and for the caregiver to give consent for how their data will be used.

The CAT developers report that it can be used in practice to identify caregivers who are at risk and in need of a formal needs assessment whilst also providing necessary support.^24^ It has been piloted for use in the United Kingdom and was found to be beneficial in mapping change over time, particularly useful in a progressive condition like MND.^25^

Policy continually calls for caregivers’ needs to be addressed, but practice still lags way behind because interventions are not integrated in existing practice but rather sit alongside it. A pilot project was needed to evaluate the relevance of the CAT intervention to family caregivers of people with MND and to service providers in a practice environment as well as the feasibility of its integration into routine practice across the caregiving experience, and not just at end of life.

## Objectives

To assess the feasibility and impact of the CAT with family caregivers of people with MND providing home-based care.

## Methods

The study ran from April 2020 to November 2021 in Western Australia. Approval was granted by La Trobe University Human Research Ethics Committee (HEC20083). Written consent was provided by all participants.

### Design

The study was a descriptive, longitudinal mixed-method design to identify caregiver support needs through data capture of the completed CAT triage tool and their experience of using the tool through individual interviews with family caregivers.

Training of the MND Advisors on the use of the CAT took place at the start of the study and follow-up meetings were scheduled regularly during the data collection period to discuss challenges with implementation.

#### Sample and recruitment

The definition of a family caregiver for this study is someone who provides care, which may be daily or intermittent, without payment to someone with MND who is expected to die during the period of caring. Adult family caregivers, registered with the MND Association of Western Australia (MNDAWA), were eligible for inclusion in the study if they were aged 18 years or above; they were a resident family caregiver (co-habiting); they were able to read and write English and they had no cognitive impairment (as determined by clinical service providers). Family caregivers were excluded from the study if deemed at the time to have very high levels of caregiver distress and participating would be an additional burden.

In their standard role, the MNDAWA Advisors regularly meet with their clients (MND patients and their respective family caregivers) either face to face in clients’ homes or at hospital clinical appointments, or over the phone. In this study, participating MND Advisors approached family caregivers, explained the study, enrolled them if interested and sought their consent to be contacted by the researcher to undertake the feedback interview at the end of the study.

#### Data collection

There were two phases to the data collection. In Phase I, family caregivers completed at least two CAT forms (typically at 6–8 weeks intervals) followed by discussions of their needs with the MND Advisors. Although it was preferable that the first CAT contact be face to face to establish rapport, the second encounter could be conducted by telephone or video call.

The intervention consisted of several steps:

- The caregiver was informed about the CAT by the MND Advisor and given time to consider participating.
- If they wished to proceed, the caregiver completed the CAT with the MND Advisor, rating each need in turn.
- The caregiver and MND Advisor discussed the ratings to clarify what the needs were and the priorities for support.
- Based on the conversation, a shared action plan was created in response to the needs.
- A date for review was agreed with the caregiver based on the type and level of need identified.

### Materials

The CAT v5 was used for the study consisting of 11 questions and an optional end-of-life question (Table 1). The tool was adapted to collect the timepoint as this was a longitudinal study, and the mode of completion as there were three options of in-person, phone or video call.

**Table 1.** CAT questions per domain.

Questions	Response format
(1) Do you currently have any needs or concerns about providing care or your own health and wellbeing? (*please circle one*)	Yes, no, unsure
**Part A: Current caring situation**	
*How much support do you need*: (2) . . .with any information about the person’s condition and how their care needs might change over time?	0 = none, 1 = low, 2 = medium, 3 = high
(3) . . .to provide any of the personal care or general daily care? (*e.g. additional support, training for lifting and handling, equipment*)	
(4) . . .to provide any emotional or spiritual care the person may need?	
(5) . . .to know who to call in an emergency, or out-of-hours, to discuss any concerns about the person?	
(6) . . .to feel involved in the decision-making and listened to by professionals about the care needed by the person (*Consider if the person requires power of attorney*)	
**Part B: Caregiver health and wellbeing**	
(7) . . .about financial, legal or work issues?	0 = none, 1 = low, 2 = medium, 3 = high
(8) . . .to take a break from caring during the day or overnight? (*e.g. sitting service, respite*)	
(9) . . .to balance your own needs with the demands of caring? (*e.g. attend own health appointments, social activities, caring for others*)	
(10) . . .to manage any feelings or worries that you may have? (*e.g. a ‘listening ear’ or having someone to talk to*)	
(11) How able do you feel you can continue providing care at the current level for the person with MND (*please circle one number on the scale*)	Likert scale of 1 = not very able to 5 = very able
*If appropriate*: Do you know the person’s wishes and preferences for end-of-life care? If yes, do they have a formal Advance Care Plan or Advance Health Directive? (*Please specify*)	Yes, no, unsure

MND, motor neurone disease.

In Phase II, caregivers’ perceptions of feasibility, acceptability and perceived support and impact from use of the CAT approach were collected *via* telephone interviews. The interview guide consisted of five questions (Table 2).

**Table 2.** Interview guide.

1. How easy or difficult was it for you to complete the CAT form? 2. Did you feel that completing the CAT was helpful in getting the support you needed? 3. Did the CAT affect what you did for yourself, for example, were there any changes for you and what you did for yourself after going through the CAT? Were there things you did for yourself after identifying what you needed? 4. Did you feel that your needs as a caregiver were acknowledged/listened to in a way that was distinct from the needs of the patient? 5. Do you think the CAT form could be improved in any way, for example, what, if anything do you feel you would change to improve the assessment process itself?

CAT, Carers’ Alert Thermometer.

The interviews were not audio-recorded, utilizing detailed field notes instead which were transcribed for analysis to identify keywords and phrases. This approach, based on the fair note methodology, has been shown to be pragmatic and useful for individual interviews when questions are relatively simple.^26^ With specific questions to be asked, our intention was to obtain caregivers’ general views on the use of the CAT, rather than undertake an in-depth study of their perceptions and experiences of their caring role.

### Analysis

Descriptive statistics of frequencies and percentages for categorical variables; means and standard deviations for variables measured on a continuous scale were used to describe the demographic characteristics of caregivers and needs as identified by the CAT and to summarize the most common examples of the action plan components implemented. Thematic analysis^27^ was performed on the interviews with the interviewer and a team member independently developing initial codes and themes which were then reviewed by the full team before agreeing on the final themes presented here. Exemplar quotes are provided for each theme from across the dataset.

## Findings

### Phase I: CAT tool implementation

Over the data collection period, 80 CAT tools were completed by 41 caregivers. Of these, 30 caregivers completed at least two CATs and are included in this study. The data from the first and last CAT completed are presented in this article.

#### Participant characteristics

There were 23 females (77%) and 7 males (23%) in the study, with a mean age of 60.6 years. Majority of caregivers were retired (40%); most were the married spouse or partner of the patient (87%) and most had finished high school (43%) (Table 3). There was significant variation in the length of time from receiving a diagnosis of MND and completing the CAT in the pilot. The median was 21 months (range >1–232 months or 19 years).

**Table 3.** Demographics of family caregiver.

Gender	*N*	%
Female	23	77
Male	7	23
Age		
Mean	60.6	
Employment status		
Full time caregiver	6	20
Working full time	5	17
Working part time (8–30 h)	7	23
Retired	12	40
Missing	2	7
Relationship to patient		
Wife/husband/partner	26	87
Son/daughter	2	7
Missing	2	7
Education		
Diploma/cert/trade	9	30
High school	13	43
University	6	20
Missing	2	7

#### Contact method and time taken

Two contact methods were used to complete the CAT during the study (Table 4). Initial CAT completions were conducted mainly by phone call (*n* = 20, 76%) with 10 conducted during a face-to-face meeting (33%). The last CAT completions were conducted equally between phone call and face-to-face (*n* = 15, 50% each). The initial CAT took a median of 40 min to complete, and the last CAT took a median of 30 min. There was wide variation in the time needed at each timepoint ranging from 5 to 90 min. At each point, over half of the CATs were completed under the average time, 55% at initial contact and 63% at last contact.

**Table 4.** Contact method and completion time.

Type of contact	First	Last
Phone	20 (67%)	15 (50%)
Face to face	10 (33%)	15 (50%)
Median time for completion (range)	40 min (5 to 90) *n* = 22, 8 missing	30 min (5 to 55) *n* = 24, 6 missing

#### Support needs identified

With a potential total need score of 27, the median scores on the initial and last CATs for each caregiver were 10 (range 1–22) and 8.5 (range 0–23), respectively. All caregivers had some level of need at the initial CAT completion. A paired sample *t*-test found statistically significant change in caregiver need level, t(29) = 2.110, p = 0.05, indicating a decrease in need from the initial CAT (*M* = 10.9, SD = 5.72) to the last CAT (*M* = 9.40, SD = 6.93).

**Figure 1.**
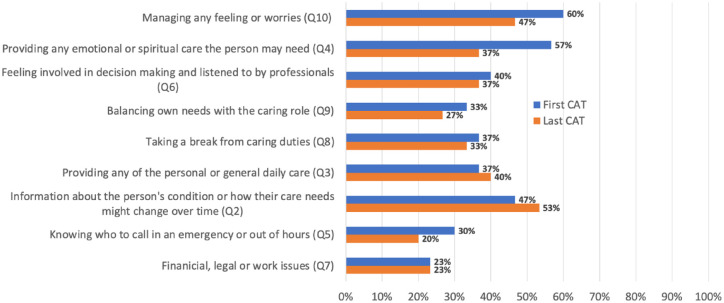
Change in medium-to-high needs identified between first and last CAT. CAT, Carers’ Alert Thermometer.

Managing feelings and worries and providing emotional/spiritual care were the most reported needs at the first CAT. Two indicators were reported to have become a greater need when completing the second CAT, most likely related to disease progression: information about the person’s condition or how their care needs might change over time and providing any of the personal or general daily care (Figure 1).

**Figure 2.**
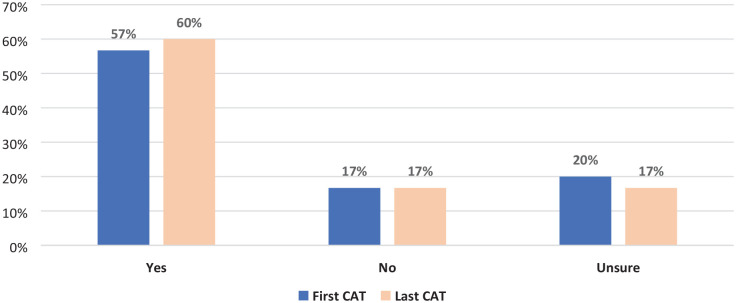
Change in the caregiver knowledge of the person’s end-of-life wishes.

Caregivers were asked if they knew the person’s wishes and preferences for end-of-life care, and if so, whether they were written down or shared in anywhere such as an advance care planning document (Figure 2). On the initial CAT, 17 caregivers did know the wishes of the person they cared for (57%) with 11 caregivers unsure or not knowing them (37%). When asked, seven of the caregivers said an advance care plan or advance health directive was in place (*n* = 7, 23%), and one said a plan was in progress at the first CAT. Knowledge of wishes improved slightly by the follow-up CAT as one caregiver changed their response from unsure to yes. Although there was no increase in reported plans in place, three additional plans were in progress.

The final question asked caregivers how able they felt to continue to provide care at the current level on a scale of 1 (not very able) to 5 (very able). There was a steady figure of 57% (*n* = 17) reporting that they felt ‘very able’ to continue and a slight increase in feeling able to cope with two caregivers moving from level 3 to 4. There was some decrease in resilience with one caregiver rating ‘not very able’ at the second timepoint suggesting an urgent need for support had been reached.

Regarding the caregiver resilience scores, of the 17 caregivers who rated themselves as ‘very able’ at the initial CAT, 12 remained there, others decreased to a rating of 3 or 4. The seven caregivers who rated themselves at 4 remained stable or moved up to 5 (*n* = 2). The four who rated themselves as 3 moved to 5 (*n* = 3) or decreased to 1 (*n* = 1) indicating a lack of resilience to continue. A Kruskal–Wallis *H* test found a statistically significant difference in caregivers’ resilience rating between the total need scores at the last CAT, *H*(2) = 16.52, *p* = 0.001. Caregivers with low to moderate needs scores are more likely to rate themselves as able to continue caring at the current level.

#### Actions to address support needs

After completing a CAT, the MND Advisors worked with the caregivers to develop an action plan to address priority areas of need identified on the CAT. The plans included actions such as providing information, supporting completion of end-of-life wishes documents, signposting to sources of support such as General Practitioners and psychologists, making a referral to the palliative care social worker and encouraging caregivers to reach out to family and friends’ support. Examples of action plans for each CAT item are listed in Table 5.

**Table 5.** Examples of actions taken or proposed by MND Advisors.

Information about the person’s condition and how their care needs might change over time	Education booklets provided such as aspects of care; appropriate referral pathways discussed; benefits of ACAT and levels of care; follow-up with OT to assist with further education
Providing any of the personal care or general daily care	Contact CATS for home alarms; advisor to make contact regarding equipment; caregiver would like training on falls prevention and lifting; discussed OT home assessment; liaise with NDIS coordinator
Providing any emotional or spiritual care the person may need	Support with starting difficult conversations; Visit GP for a mental health plan. Discussed benefits of speaking with Psych; patient and caregiver to complete AHDs with their children in coming weeks
To know who to call in an emergency, or out-of-hours, to discuss any concerns about the person	My Health Direct information line for advice out of hours, phone ambulance for immediate health concern; provided contact list for out of hours
To feel involved in the decision making and listened to by professionals	Advisor to advocate support with NDIS planner; reassured caregiver advocacy with health professional as available through MNDAWA
Financial, legal or work issues	Created a spreadsheet that has all the information needed, that is, passwords, account numbers, etc.; wanting to sort out AHD/ACP now – referral to palliative care social worker; consider accessing hardship utility grant
To take a break from caring during the day or overnight	Increased caregiver hours in NDIS plan; respite options to be increased; referral to palliative care volunteer service
To balance your own needs	Exercise; yoga; mindfulness; start swimming; delegate to appropriate family members; start making appointments for herself (i.e. hair, etc.); engage more regular FaceTime visits with friends interstate
To manage any feelings or worries you might have	Referral to MNDAWA grief counselling; reached out to a counsellor which has helped put things in perspective; feels well supported by their community and allied health team; meets with bible/church group

ACAT, Aged Care Assessment Team; ACP, advance care planning; AHD, advance health directive; CATS, Communication and Assistive Technology Service; MND, motor neurone disease; MNDAWA, MND Association of Western Australia; NDIS, National Disability Insurance Scheme; OT, Occupational Therapist.

### Phase II interview findings

On submission of a second CAT, caregivers were contacted for a follow-up interview to ascertain the effect of the CAT on supporting them in their caring role. Of the 30 caregivers from Phase I, 26 were invited to participate as the MND Advisor had requested no contact for 4 of the caregivers due to their individual circumstances including the death of 1 person being cared for. Seventeen caregivers took part in an interview as two caregivers declined, and seven caregivers did not respond to multiple attempts to undertake the interview. The key themes are presented below with exemplar quotations.

#### Theme 1: Suitability and acceptability of the CAT

Participants were asked about the ease of completion of the CAT. Many comments received indicate that the CAT was acceptable to caregivers and the process of completing it was easy and uncomplicated.

Found it relevant with pertinent questions – easily understood what the questions were asking; all made sense. (ID10)

Lack of time for one caregiver, in a life dominated by caring, initially posed difficulties in finding an appropriate opportunity to complete the CAT.

The form wasn’t difficult; what was difficult was allocating the time. Advisor initially gave it to me to complete on my own; in the end they sat down with me to complete which allowed me to prioritise it and complete it. (ID16)

One caregiver regarded the process as more challenging and found it ‘*confronting; thinking deeply about things*’ (ID12).

##### Sub theme: Continuing use of CAT as a tool to support caregivers

The CAT was regarded as a ‘brilliant idea’ (ID16) which should be repeated on a regular basis; it was seen as an important means of ensuring that caregivers’ needs would continue to be assessed and addressed as time progressed and the caring situation changed:

Caregivers should fill it in regularly (e.g. every 6 months) to keep everyone they are involved with updated. (ID20)

#### Theme 2: Genuine focus on caregivers

Participants spoke positively about how the CAT enabled the focus of conversations with MND Advisors to be specifically on the caregivers, even if only for a brief time.

This was a conversation all about me; nothing to do with my [patient]. (ID17)

Don’t usually think about yourself; focussed on [patient]. (ID25)

Yes; helped me identify what was wrong in my life and how to support myself. (ID37)

Gave me the tools and prompted me to find the information and support I needed. (ID13)

Often, caregivers were unaware of what they might be able to do to help themselves; there was an overwhelming sense that the CAT definitely facilitated the process of identification. Caregivers were able to clarify where problems existed and work through solutions.

Was amazing really; didn’t realise it would solve all my problems, it helped me identify what was wrong in my life and how to support myself. Brilliant outcomes using this tool; helped to point out what I needed to do in order to help myself. Helped me get clarity – calm, peace. (ID37)

Helped me to develop tactics. (ID4)

Additionally, the CAT was a reminder to caregivers that they needed to consider their own health and well-being and make changes to what they did for themselves, if they were to be able to continue in their demanding caring role.

Cemented thoughts about needing to look after myself. (ID11)

I have made changes for my own health. (ID25)

##### Sub-theme: Enhanced caregiver/advisor relationship

As a direct consequence of completing the CAT, caregivers reported improved relationships with MND Advisors.

Opened doorway for 1:1 conversation with Advisor . . . which has had a continuing positive impact on our relationship. (ID17)

A number of participants expressed the view that completing the CAT had enabled them to discuss issues which they would otherwise not have considered raising with the MND Advisor. Working together with Advisors in this way was very much appreciated by caregivers.

Enabled the Advisor to ask questions related to the form that I wouldn’t have thought about and I think just reading it on my own I still wouldn’t have thought about it – allowed for us to dig a little deeper and helped that we already had a good relationship. (ID16)

Form was good; took me a while to get into it because I wasn’t ready for it but glad I did it; good discussions to have with my Advisor and brought out stuff to be aware of and to do. That side was really good. (ID15)

#### Theme 3: Relevance/improvements

The items included on the CAT were viewed as relevant to caregiver circumstances.

Covers all needs; recognising everyone’s needs are different. Fine as is. (ID25)

However, suggestions to improve the process were offered; some were concerned with the CAT itself.

Maybe there is a need for some more warm and fluffy question that could possibly put participants at ease but I’m not good at the sort of thing therefore I’m not sure what format you could use. (ID3).

Have the plan as a guide that’s printable and top of mind, i.e. section 4 – have this as a separate page and encourage people to print out and put somewhere prominent as a reminder. (ID10)

Other comments referred to the timing of introducing it to caregivers and the procedure adopted.

Thought the process could be improved by introducing the CAT form earlier, i.e. once someone is diagnosed (in the very beginning). At the onset you have no idea – so many new terms, e.g. didn’t understand terms like what to do in an emergency, balancing caregiver needs. You are so caught up in the diagnosis that you are not thinking ahead. Completing the CAT prompted me to find and seek relevant information much earlier – should be part of the diagnosis information package to prompt thought processes. (ID13)

Have the Advisor sit down and complete with you – makes you prioritise completing the form and also to think about it more. If we had done them earlier (in the diagnosis) it would have helped alleviate/prevent some of the issues. (ID16)

Other suggestions, triggered by the CAT conversation on preferences for end-of-life care, were about improvements in palliative care information and education:

More palliative care information should be provided. (ID11)

No preparation for dying with dignity; everyone [all providers] should be educated in how to give good palliative care to family. (ID11)

Additionally, caregivers felt they would benefit from having access to further information, scenarios and advice associated with the CAT that they could access at any time.

No specific changes to the form suggested as it is very clear; however, suggested that it should be located on the MND Association website as a form with hyperlinks to examples/case studies and advice relevant to each question because some questions you might think aren’t relevant but actually are; sometimes you think your question is a stupid one, so you don’t ask. (ID13)

Whilst the majority of participants had a positive experience with the CAT, one caregiver reported that their MND Advisor was less than enthusiastic about introducing it, which affected their view of its usefulness.

MND Advisor said it was ‘a waste of time’ and ‘won’t accomplish much’. (ID14).

### Phase III: Family caregiver online support and information toolkit

Phase III arose as a response to feedback from the interviews and staff debriefs that suggested having relevant information, such as resources from formal and informal networks, which was easily accessible, would support both caregivers and staff. The creation of the toolkit provides the opportunity to develop evidence-informed staff with the confidence and understanding of the benefits of regular screening and guide how to proceed once the CAT had been completed. This gave rise to a WA-specific Family Caregiver Online Toolkit (www.mndawa.asn.au/family-carer-support).

**Figure 3.**
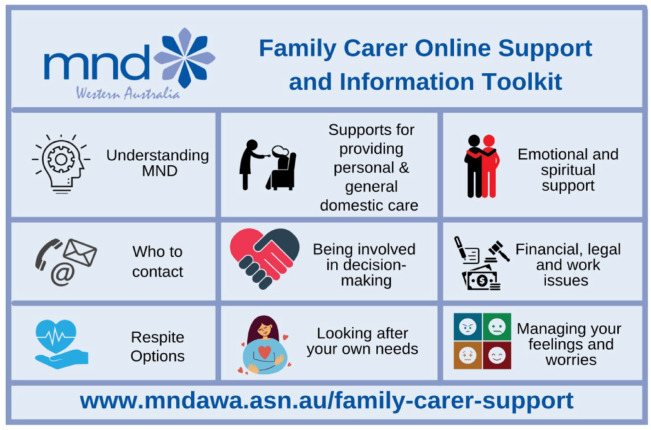
Areas of the family caregiver online support and information toolkit.

The toolkit has nine separate sections, which are based on the key areas of caregiver need identified within the CAT. For each toolkit section, there is: general information about the topic; useful links for resources and support; experiences/stories to help caregivers relate to why they might need some information on that topic, based on feedback/stories from MNDAWA caregivers. The toolkit supplements the face-to-face and phone support that MNDAWA already provides and allows caregivers a place to look for information in their own time. The toolkit was depicted in a fridge magnet, which was posted to all 190 registered caregivers. It had the link to the website page as well as a picture of the nine toolkit sections (Figure 3).

Feedback was sought from caregivers who checked the toolkit using the online feedback form. Caregivers found the toolkit helpful due to its 24/7 accessibility, clear information, comprehensive topics and use of practical examples.

I feel like it will be of great benefit; it’s not too wordy and doesn’t go into minute detail but has follow up sites for those who need or would like more detail as well as sites for practical help.

I like how it has both details of the disease, MND, and ways to help and support those with it, e.g. OT, equipment, NDIS, ACAT, hospital teams etc and then goes onto support for the caregiver.

I found the practical examples of people seeking solutions to their situation helpful; it gave me a feeling of not being alone.

I feel it will be a valuable tool to have and, being online, is available 24 hours a day as well as being able to be updated/amended as needed.

Whilst the toolkit was developed to address caregivers’ requests for advice in relation to the topics covered in the CAT, it was also intended for use by staff working with caregivers. The toolkit enables staff to access up to date information on evidence-based areas of caregiver need to help guide them in their deliberations with caregivers, as some staff struggled to assist caregivers in terms of information and signposting.

## Discussion

This pilot study has explored the feasibility and relevance of the CAT tool in home-based care from the perspective of family caregivers of people with MND. The sustainability of such an initiative goes beyond what caregivers want, to what service providers are willing to do, and the consideration of collaborative models of care, as discussed below.

### Effect on caregivers

Participants found the CAT approach acceptable, relevant and easy to use. The CAT was able to identify need and resilience to continue caring in those who took part through its systematic approach to reviewing evidence-based areas of caregiver need. A key outcome in this study, and reflective of earlier findings, was the ability of the CAT to facilitate ongoing assessment of caregiver needs, vital given the often-rapid deterioration associated with MND,^25^ and crucial to address calls for regular monitoring of caregiver welfare.^28^ Repeating the CAT and appropriate provision of support was found to reduce the self-reported level of need and improve resilience suggesting that repeating the CAT at regular intervals, depending on the level of need and resilience, could provide benefits for caregivers from an early stage. Participants suggested introducing the CAT at the time of the diagnosis would be most useful. Given the large variation of the time between diagnosis and completion of the CAT for many participants in this pilot, this recommendation from caregivers is key for early identification of their needs and the support required for both the caring situation and carer’s own health and well-being.

During the CAT pilot, advisors may have invited caregivers who were not experiencing high levels of stress to take part in order to avoid burden. This gatekeeping approach meant it was anticipated that the CAT scores may not be overly high and there may be limited change. The findings, however, show that all caregivers had some level of need when completing the initial CAT and some identified a high level of need with scores ranging from 1 to 22 (potential total score is 27). This highlights that the level of needs for caregivers cannot be assumed by others and that they require, and deserve, systematic and supportive ongoing review of their needs before challenges reach a crisis point.^20^ There was a statistically significant change in the overall CAT level of need from initial to last CAT with reduced need across many of the indicator questions. The two indicators which had increased were Q2 ‘information about the person’s condition or how their care needs might change over time’ and Q3 ‘providing any of the personal or general daily care’. These two indicators reflect a change in the level of care required to support the cared for person as their illness progresses, and that caregivers need more information once the initial response to the news of the diagnosis had passed.

### Service providers’ concerns

It has been noted in previous studies that there can be some gatekeeping and pushback by service providers when it comes to which caregivers will meet the criteria for screening and assessments.^29^ It is also acknowledged that clinicians have limited time to screen for caregivers’ need and often have a lack of awareness of available resources to support them.^30^ This can lead to inequalities in the provision of support and care.

For any tool to become an integrated part of care, it is important for there to be service provider support for implementation factoring in the time resource required and an appropriate education and support structure for those involved. Previous work has suggested that there can be reluctance from staff acting as assessors with a tool like CAT, due to concern they will be asked to respond and resolve lots of needs, a lack of confidence to manage conversations and a lack of awareness of available resources to signpost the caregiver to.^24,25^ Other research concluded that the more assessments were administered, the more insight was gained and the more supportive health professionals became of this systematic approach. In addition, the longer time the health professional worked in this field, meaning the more experienced, the more supportive they were.^31^

Service providers can also question the use of a formal, systematic tool which can be a barrier to introducing any caregiver tool into routine practice. An example in this study was one advisor telling a caregiver that it ‘*won’t accomplish much*’. Appropriate training can empower the assessors to become more knowledgeable about available support and reconsider the need for any restrictive criteria which might limit use of the CAT or other tools.^25^ For assessors to utilize tools such as the CAT successfully, they need to feel confident having discussions on sensitive topics, such as end-of-life wishes, and knowledgeable about where to signpost caregivers to clear, relevant information. This can be fostered through appropriate training programmes, which take on board the different backgrounds of the staff as their experience will influence their practice. Essential sessions include communication skills, how to handle challenging or upsetting conversations, and how to bring them to a close providing the caregivers with a clear sense of being heard and what is possible regarding their priority needs. In this study, it was apparent that a toolkit of information and resources would be helpful to assist caregivers and service providers improve their knowledge of what is available in their community in terms of formal and informal support. Furthermore, the detailed description of implementation of the CAT in this article can be incorporated as an educational resource. To this end, funding has been granted for the national training and roll out of the CAT in Australia.

### A needed shift to a Compassionate Communities Collaborative model of care

Assessing needs is a first step in acknowledging areas where caregivers need support. In this study, caregivers have reported their first priority needs being ‘managing feelings and worries and providing emotional/spiritual care’. Similarly in other studies, caregivers are usually left wanting in the psychosocial support and improvement in their social connectedness and reduction in their social isolation.^32^ This lack of support from formal and informal networks was highlighted in this study by comments such as: ‘*the reality is really bad and supports are not there; we live it every day, every minute; It’s just long and depressing and lack of family support; no insight [of various providers] into the psychology and devastation of the prognosis*’ (ID11).

This is usually best addressed within the realm of support from caregivers’ naturally occurring or facilitated social networks, which can be bolstered through Compassionate Communities Connectors models of care.^33,34^ Connectors, who are community volunteers, built the capacity of caring networks around families in need, whether from the naturally occurring networks or externally facilitated ones. The range of support given through such models is generally far wider than the domains used in any carer assessment tool. The impact of improved social connectedness and the range of support provided by the caring network raises the question as to whether a high carer needs assessment score is a marker of lack of community networks, rather than the increased need for professional services. Therefore, rather than looking to more professional services to provide caregiver support, a first step might be to ensure that enhancement of supportive networks takes place.^14,34^ To this end, Advisors have explored with caregivers in a few instances the viability of their family and social networks to provide support (Table 5).

Building the capacity of friends by ensuring they are skilled in when and what to offer to support caregivers can provide symbiotic benefit for both friend and caregiver as one participant identified: ‘*If there is one thing I have learnt from our MND journey is that friends want to help and you may not need anything at the time, however, you just need to accept what they offer gratefully as they feel better to have done something*’ (ID3). Indeed, accepting help is the first step towards building and skilling supportive networks around the caregiving family and into bereavement.^33^ MND Associations can act as enablers for formal and informal networks to work together to provide a platform of psychosocial, emotional and existential support that is sustainable in people’s own communities.^35^

## Limitations

This pilot was designed as a practical intervention with quantitative and qualitative evaluation components to assess feasibility. The interviews were brief and focused on eliciting the overall perception of the benefits and challenges. They were not intended to provide data for an in-depth study of personal insights. As interviews were only completed with 17 of the 30 caregivers in the study, their views may not reflect those of the remaining participants. There was some gatekeeping regarding which caregivers were invited to participate and this may not reflect the level of need in the broader caregiver population.

## Conclusions

Despite global emphasis on supporting caregivers,^9,10^ assessment of their needs remains haphazard. By devoting time specifically to discussing the caregiver’s situation, use of the CAT acknowledges the importance of their role and the impact that caring can have on their health and well-being. Use of the CAT would promote a more systematic and consistent service practice for caregivers and inform policy in home-based care. MND Associations are ideally placed to foster a Compassionate Communities Connectors model of care, as they can connect both professional and community resources in a way that health professionals alone, or community actors alone, cannot. However, this requires MND Associations to include caregiver support as an integral part of their role, access appropriate training for their staff and engage community volunteers with MND lived experience to bridge the gap in practical and psychosocial support.

## Declarations

**Ethics approval and consent to participate:** The study was conducted according to the guidelines of the Declaration of Helsinki and approved by La Trobe University Human Research Ethics Committee (HEC20083). Participants provided written informed consent prior to the start of data collection.

**Consent for publication:** XXX.

**Availability of data and materials:** Ethical approval precludes the data being used for another purpose or being provided to researchers who have not signed the appropriate confidentiality agreement. Specifically, the ethical approval specifies that all results are in aggregate form to maintain confidentiality and privacy and precludes individual level data being made publicly available. All aggregate data for this study are freely available and included in the paper. Interested and qualified researchers may send requests for additional data to Samar M. Aoun at samar.aoun@perron.uwa.edu.au.
